# Anodal Transcranial Direct Current Stimulation Induces High Gamma-Band Activity in the Left Dorsolateral Prefrontal Cortex During a Working Memory Task: A Double-Blind, Randomized, Crossover Study

**DOI:** 10.3389/fnhum.2019.00136

**Published:** 2019-04-24

**Authors:** Takashi Ikeda, Tetsuya Takahashi, Hirotoshi Hiraishi, Daisuke N. Saito, Mitsuru Kikuchi

**Affiliations:** ^1^Research Center for Child Mental Development, Kanazawa University, Kanazawa, Japan; ^2^Department of Neuropsychiatry, University of Fukui, Fukui, Japan; ^3^Department of Biofunctional Imaging, Preeminent Medical Photonics Education & Research Center, Hamamatsu University School of Medicine, Hamamatsu, Japan; ^4^Department of Psychiatry and Neurobiology, Graduate School of Medical Science, Kanazawa University, Kanazawa, Japan

**Keywords:** tDCS, working memory, DLPFC, MEG, phase-amplitude coupling, n-back task, color

## Abstract

Transcranial direct current stimulation (tDCS) has been shown to have mixed effects on working memory (WM) capacity in healthy individuals. Different stimulation paradigms may account for these discrepancies, with certain features being favored. To determine the effect in the context of anodal tDCS, we investigated whether anodal tDCS induced cortical oscillatory changes during a WM task. Specifically, we tested whether anodal offline tDCS over the left prefrontal cortex (PFC) enhances WM capacity by modulating the oscillatory activity in the left dorsolateral PFC (DLPFC) using magnetoencephalography (MEG). This study employed a double-blind, randomized, crossover design, in which 24 healthy right-handed participants conducted MEG recordings during a 3-back task after administration of 2 mA tDCS or sham stimulation as a placebo. Our results showed that the effect of tDCS did not appear in the behavioral indices—WM accuracy (*d*′) or reaction time (RT). From the results of the time-frequency analysis, significant event-related synchronization (ERS) in the high-gamma band (82–84 Hz) of the left DLPFC was found under the tDCS condition; however, ERS was not correlated with WM capacity. Furthermore, we calculated the modulation index (MI), which indicates the strength of phase-amplitude coupling (PAC). tDCS significantly decreased MI of the left DLPFC, representing the theta-gamma PAC during the n-back task using color names as verbal stimuli. Our results suggest that although tDCS increased the gamma-band oscillation indicating greater neural activity in the left DLPFC, it did not lead to an improvement of WM capacity; this may be due to the inability of gamma-band oscillation to couple with the task-induced theta wave. WM capacity might not increase unless theta-gamma PAC is not enhanced by tDCS.

## Introduction

Working memory (WM) permits the maintenance of perceived information over a short period of time. WM has specialized buffers, a phonological loop and visuo-spatial sketchpad, and the central executive, which represent executive function (Baddeley and Hitch, 1974; Baddeley, 2012). Executive function has been a focus of recent research as it serves as an attention controller that allocates and coordinates attentional resources for a variety of cognitive tasks (Osaka et al., 2007). Executive function is needed to solve complex (“frontal lobe”) tasks and is thought to comprise three subcomponents—shifting, updating, and inhibition (Miyake et al., 2000). Shifting describes the flexibility of switching between tasks or mental sets, updating is the ability to monitor and rapidly add to or delete WM contents, and inhibition is the ability to deliberately override dominant or prepotent responses (Miyake and Friedman, 2012). For example, the *n*-back task, which is frequently used to measure WM capacity, relies more heavily on concurrent updating ability than it does shifting (Kane et al., 2007; Snyder et al., 2015). Neuroimaging studies suggest that executive functions are located in the prefrontal cortex (PFC), cingulate cortex, and parietal cortex (Baddeley, 2003; Niendam et al., 2012). In particular, activation of the left dorsolateral PFC (DLPFC) has been observed in tasks that require executive function (Smith and Jonides, 1999). In electrophysiology, the relationship between WM and brain rhythms has been studied (Klimesch, 1999). Electroencephalography (EEG) and magnetoencephalography (MEG) studies have frequently reported event-related oscillatory changes, which are considered to represent the increase or decrease in synchronous activity of neuronal populations. When frequency-specific changes of the ongoing oscillatory power occur, the increase or decrease of power is called event-related synchronization (ERS) or desynchronization (ERD), respectively (Pfurtscheller and Lopes da Silva, 1999). Some studies have reported prominent theta power increases over frontal regions during various WM tasks (Ishii et al., 1999; Jensen and Tesche, 2002; Hsieh and Ranganath, 2014). Task-dependent theta band oscillations recorded over the frontal cortex have been shown to increase with memory demand (Jensen and Tesche, 2002). Furthermore, higher frequencies have also been shown to contribute to WM function. Inhibitory gamma-aminobutyric acid (GABA) neurons in the DLPFC mediate the synchronization of pyramidal neurons at the gamma frequency; accordingly, patients with schizophrenia, where synthesis of GABA is decreased, frequently present with WM deficits (Lewis et al., 2005). An integrated study using EEG and magnetic resonance spectroscopy confirmed that *in vivo* GABA measures, gamma-band oscillations, and WM capacity were tightly correlated (Chen et al., 2014).

Recently, advancements have been made in studies aimed at improving WM capacity through non-invasive stimulus methods (Steinberg et al., 2018). Transcranial direct current stimulation (tDCS) is a widely used technique for non-invasive brain stimulation, which is a subset of transcranial electrical stimulation (tES) methodology (Nitsche and Paulus, 2011). During its initial study, the effect of tDCS on motor function was investigated. tDCS over the motor cortex depends on its current polarity, with research suggesting that anodal tDCS increases excitability of the motor cortex, whereas cathodal tDCS decreases excitability (Nitsche and Paulus, 2000). The mechanism of excitability change caused by tDCS has been studied electrically and pharmacologically. One animal study found that anodal currents to the cortical surface depolarized pyramidal neurons, whereas cathodal currents hyperpolarized them (Purpura and McMurtry, 1965). In a human study, cortical excitability continued even after cessation of current stimulation; however, this aftereffect was blocked by an NMDA receptor antagonist (Nitsche et al., 2003). In addition, tDCS extending over a few minutes led to LTP-like plasticity, which could spread to other cortical and subcortical regions (Polania et al., 2012). Taken together, it is thought that direct current has a modulation effect on cortical plasticity (Stagg and Nitsche, 2011). Oscillatory changes caused by tDCS was also reported in some articles. Anodal tDCS applied to the occipital region has been found to elicit gamma band ERS in the visual cortex (Hanley et al., 2016; Wilson et al., 2017). Since tDCS has been shown to modulate brain activity, enhancement of cognitive function has also been studied. Among cognitive functions, of particular interest has been the acute influence of tDCS on executive functions (Strobach and Antonenko, 2017). Many studies have stimulated the left PFC, which is the core brain region involved in cognitive function (Santarnecchi et al., 2015). F3, the left prefrontal site in the international 10–20 system, is located approximately above the left DLPFC and is the primary candidate for placing an anode during tDCS. Anodal tDCS over F3 has been shown to improve WM capacity, compared to sham, cathodal tDCS, and anodal tDCS over the motor cortex (Fregni et al., 2005). The effect of polarity of direct current stimulation on cognitive function is difficult to study. From a meta-analysis study, the anodal-excitation effect is commonly found in cognitive studies, but cathodal-inhibition effects are unclear (Jacobson et al., 2012).

However, while positive effects of tDCS on WM capacity have been reported, negative results have also been found. For example, tDCS over the left DLPFC had no effect on n-back accuracy, reaction time (RT; Mylius et al., 2012; Hoy et al., 2013; Hill et al., 2018), or Wechsler Adult Intelligence Scale, Fourth Edition (WAIS-IV) scores (Sellers et al., 2015). There are several possible reasons for these differences in results including stimulation site, polarity, current, cathode location, length of stimulation, and online vs. offline stimulation (Medina and Cason, 2017). One review reported that offline anodal tDCS applied to healthy participants improved WM accuracy and RT, whereas online did not (Hill et al., 2016). Thus, the impact of tDCS on WM capacity is still unclear and its neural basis should be better defined, ideally using the commonly used n-back task. Gamma oscillations are the key to interpreting the effect of anodal tDCS, WM capacity, and the left DLPFC.

Thus, we selected a stimulation method with a high possibility of improving WM capacity and investigated tDCS-induced neural activity changes. tDCS should be effective with anodal stimulation and an offline paradigm. Here we report the effects of tDCS on behavioral and neurophysiological state. We hypothesized that anodal offline tDCS over F3 will enhance WM capacity by modulating the oscillatory activity in the left DLPFC using MEG. If tDCS effectively stimulates the left DLPFC, oscillatory changes should occur during a task which elicits strong activation in that region. WM capacity was measured by the 3-back task. The n-back task is a continuous performance test used to estimate WM capacity (Rosvold et al., 1956; Haatveit et al., 2010). The task requires participants to monitor whether the current stimulus is the same as the one presented *n* trials before—where *n* is a predefined number, usually 1, 2, or 3. As we assessed the effects of tDCS on WM performance, floor and ceiling effects should be avoided. For healthy young adults, the 2-back task can be performed easily (Ikeda and Osaka, 2007) and, without special training, the 4-back task is difficult (Buschkuehl et al., 2014); accordingly, the 3-back task was considered suitable to study the effects of tDCS on WM performance. In a previous fMRI study (Ikeda and Osaka, 2007) performed with right-handed participants, the 2-back task using verbal stimuli (Word condition) increased activity in the left PFC, which is an important region for verbal WM (Smith et al., 1998). In addition, presentation of visual color stimuli that belong to the same color category (Within condition) activates the right PFC, whereas using highly codable color stimuli (Cross condition) has intermediate properties among the other two conditions. These results indicate that the items to remember in the n-back task could bias the balance between the left and right hemispheres of activation areas according to participant’s dominant language hemisphere. If tDCS activates verbal WM and updating ability together, WM capacity and/or neural oscillations would be enhanced in the Word condition.

## Materials and Methods

### Participants

Twenty-four healthy adult male students (mean = 21.3 years old, SD = 1.26) were recruited from Kanazawa University and participated in this experiment. All participants were right-handed, which was assessed by the Edinburgh Handedness Inventory (Oldfield, 1971). They had normal or corrected-to-normal vision. The Farnsworth Dichotomous Test for color blindness (Panel D-15) was used to assess color vision. One participant had a suspected case of Deuteranopia, however, he passed the color discrimination test described later. Participants were native Japanese speakers with normal hearing and had no medical or family histories of neurological or psychiatric disorders. Full IQ scores (mean = 108.4, SD = 5.83) were estimated using the Japanese version of the National Adult Reading Test (Matsuoka et al., 2006). Participants agreed to participate in this study with full knowledge of the experimental nature of the research. Each participant provided written informed consent prior to participation. The Ethics Committee of Kanazawa University approved this study, which conformed to the tenets of the Declaration of Helsinki.

### Experimental Design

**Figure 1:**
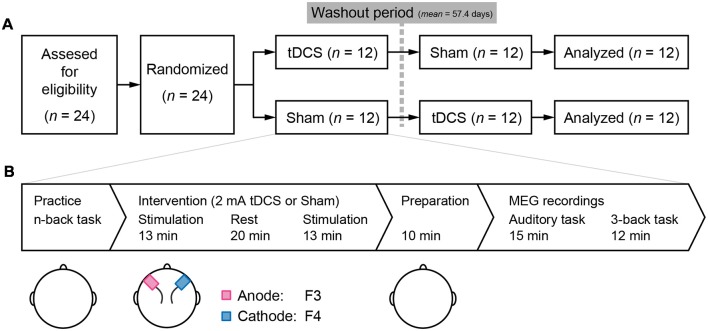
**(A)** Study design: a double-blind, randomized, crossover design was employed. Twenty-four participants were recruited and randomly assigned to receive either transcranial direct current stimulation (tDCS) or Sham stimulation during the first session. After a washout period of at least 1 month, the second session was conducted. **(B)** Task flow of the experiments in each session: practice of the n-back task was conducted in the order of 1-, 2-, and 3-back conditions. tDCS or sham stimulus as a placebo was administrated. Two sponge electrodes, anode and cathode were on the F3 and F4 according to the international 10–20 system, respectively. Electrodes were removed and preparation for magnetoencephalography (MEG) recordings in a shielded room was initiated. The first MEG task was an auditory task reported in Miyagishi et al. (2018). The 3-back task was started approximately 25 min after the end of stimulation.

The study employed a randomized double-blind, controlled placebo, crossover design that included washout period of at least 1 month (mean = 57.4 days, SD = 25.9). Initially, all participants were randomly assigned to either the tDCS-Sham or Sham-tDCS group. At the beginning of each testing day, participants performed a practice session of the 1-, 2-, and 3-back task. Next, participants were administered tDCS or sham stimulation with 20-min rest between two 13-min stimulation. After the stimulation, participants were prepared for MEG recordings and received a 10-min explanation of the procedure. Following a 15-min auditory task (Miyagishi et al., 2018), we measured the MEG signal to investigate the neural effects of tDCS on the 3-back task (Figure 1A). After all the experiments were finished, participants conducted a color naming task and a color discrimination task to check that all color stimuli in this experiment were recognizable and discriminable.

### tDCS

A direct current was induced through two saline-soaked surface sponge electrodes (5 × 7 cm) and delivered using a battery-driven, constant current stimulator (DC-STIMULATOR Plus, neuroConn GmbH, Germany). The anode electrode was placed over F3, and the cathode electrode was placed over F4 (see the international EEG 10–20 system) during stimulation (Figure 1B). Participants received the stimulus twice before MEG recording, and the duration of a stimulation was 13 min at a current strength of 2 mA to maximize the aftereffects of stimulation (Monte-Silva et al., 2013). During the sham stimulation, electrodes were also attached to the participant, but the current was only delivered during the first 10 s, which prevented the participants from noticing the absence of electrical stimulation.

### n-Back Task

**Figure 2:**
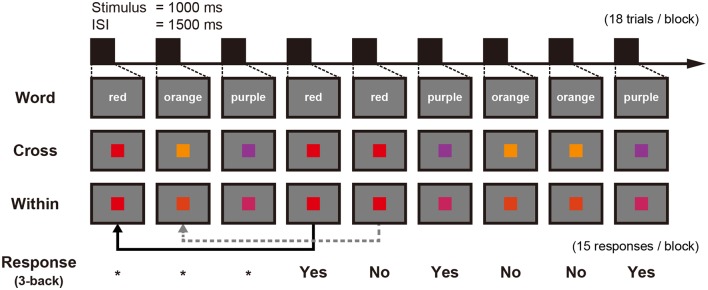
Schematic figure of an experimental block showing three conditions and the corresponding 3-back responses: stimuli for the Word condition are represented here in English instead of Japanese Kana used within the tDCS-MEG study. *Means that no response is needed.

A block in each n-back task contained 15 trials to respond. In the 3-back condition, a block contained 18 trials as the first three trials were only for encoding (Figure 2). Each stimulus was presented for 1,000 ms followed by a 1,500 ms interstimulus interval (ISI). Participants had to respond with their right index or middle finger depending on whether the stimulus was the same or different from the one presented in three trials previously, using a response pad (LUMINA LU400-PAIR, Cedrus Corporation, San Pedro, CA, USA). The percentage of both “same” trials and “different” trials was 50% within each condition. WM accuracy was measured using *d*′ which is calculated from hit rate and false-alarm rate (MacMillan and Creelman, 2004) and RT was defined as the time from a stimulus presentation to button press.

All participants had practice sessions using capital letters (from A to H) that were not presented in the MEG recording session to confirm that they understood how to perform the n-back task. At first, participants completed a 1-back and 2-back condition until they achieved an accuracy greater than 85%. Following the successful completion of these tasks, a fixed-length practice session of the 3-back task and six blocks were conducted. These practice sessions were conducted before tDCS or sham stimulation in the both days.

In the MEG recordings, we employed verbal (color word) or visual (color rectangle) stimuli as items to remember during an n-back task. In the Word condition, Japanese words describing the color name were in white (Meiryo font, 36 point). The color coordinates of stimuli are listed in Supplementary Table S1. Visual stimuli were presented on the screen in front of a participant using a liquid crystal projector (IPSiO PJWX6170N, Ricoh Company Ltd., Tokyo, Japan). All stimuli were controlled through Presentation (Version 13.1, Neurobehavioral Systems, Berkeley, CA, USA) running on Windows XP. The luminance and chromaticity of color stimuli were measured by a luminance and color meter (CS-200, Konica Minolta, Japan). The size of color stimulus was 5.6° × 5.6°, and the neutral gray background field was 24.1° × 21° (width × height). An optical sensor connected to the MEG system was attached outside of the background field, which generated a trigger signal synchronizing with the start time of visual stimulus presentation.

### MEG Recordings

Magnetic fields were measured using a 160-channel whole-head-type system (MEGvision PQA160C; Ricoh Company, Ltd., Kanazawa, Japan). Sensors were configured as first-order coaxial gradiometers with a baseline of 50 mm; the diameter of each coil of the gradiometers was 15.5 mm. Magnetic fields were sampled at 2,000 Hz per channel with a 500 Hz low-pass filter. Using a Signa Excite HD 1.5T system (GE Yokogawa Medical Systems Ltd., Milwaukee, WI, USA), we obtained a T1-weighted structural image with spherical lipid markers placed at the five MEG fiducial points to enable us to superpose the MEG coordinate system on the MRI data. A T1-weighted image consisted of 166 sequential 1.2 mm-thick slices with a resolution of 512 × 512 points within a field of view of 261 × 261 mm. The cortex surface was reconstructed using Freesurfer software (version 5.3^1^).

### Data Analysis

Behavioral data processing and analysis were performed using R software (version 3.5.1^2^). Each dependent variable, *d*′ for accuracy and RT for speed, was analyzed using a two-way repeated measures analysis of variance (ANOVA), with Intervention (tDCS, Sham) and Condition (Word, Cross, Within) as the within-subject factors.

MEG data processing and analytical procedures were performed using Brainstorm software (Tadel et al., 2011) ran on MATLAB^®^ (version R2016b, The MathWorks, Natick, MA, USA). Four noisy channels were eliminated from the analysis. Eye-movement and cardiac artifacts were removed using the signal-space projection (SSP) method. Segments that included head movement or muscle artifacts detected in a visual inspection or in the automatic processing procedure in Brainstorm, were discarded. Next, data were filtered using band-pass (0.5–100 Hz) and notch (60 Hz) filters. The epoch was defined as −1,000 to 3,000 ms relative to the visual stimulus onset (0 ms), followed by selecting correctly encoded trials.

We estimated the signal source using the anatomical cortical surface data of each subject tessellated with 15,000 vertices. The lead field was then computed using the overlapping spheres algorithm. The inverse solution was calculated for each session through the linearly constrained minimum variance vector beamformer. A noise and data covariance matrix were calculated based on the MEG recordings obtained during the −100 to 0 ms, and 0–2,350 ms time windows of every epoch within a session.

Two regions of interest (ROIs: L/R DLPFC = Rostral Middle Frontal) were determined based on the Desikan-Killiany atlas (Desikan et al., 2006) implemented in Freesurfer. Signals were taken from the first mode of the principle component analysis decomposition of the signals within each ROI. A time-frequency analysis was conducted using a multi-taper convolution method with the Hanning window (0.3 s). The Event-related spectral perturbation (ERSP) represents the event-related percent changes in signal magnitude relative to a prestimulus baseline period (from −400 to −100 ms). To compare the neural activation under the tDCS and sham conditions, we conducted paired-sample permutation *t*-tests on the data, which contained the three following dimensions: ROI (left/right), time (–500 to 2,500 ms), and frequency (1–100 Hz). The statistical threshold was set at *p* < 0.05, two-tailed, with a false discovery rate (FDR) correction. The additional analysis on the gamma-band power, which was significantly affected by tDCS, was conducted using a two-way repeated measures ANOVA, with Intervention (tDCS, Sham) and Condition (Word, Cross, Within) as the within-subject factors. Furthermore, a correlation analysis was performed to explore the correlation between gamma-band oscillations and WM capacity (*d*′).

## Results

**Figure 3:**
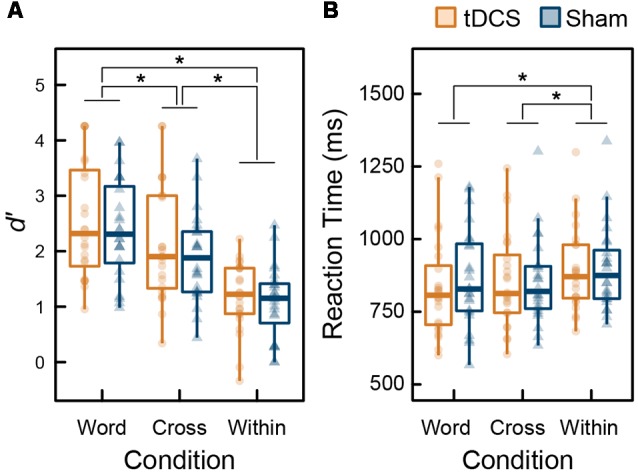
Box plots with individual participant data of **(A)** *d′* and **(B)** reaction time (RT): stars denote significant difference at *p* < 0.05.

Figure 3 summarizes the behavioral data of the 3-back task during MEG recordings. To assess the ceiling or floor effect on WM capacity, we calculated skewness of *d*′ (range: −0.67 to 0.47). No highly skewed distribution was found, and thus the ceiling or floor effect was not observed. From the results of the ANOVA performed on *d*′ data, the main effect of intervention was not significant (*F*_(1,23)_ = 1.140, *p* = 0.297, $\eta_{\text{p}}^{2}$ = 0.047), the main effect of condition was significant (*F*_(2,46)_ = 58.038, *p* < 0.001, $\eta_{\text{p}}^{2}$ = 0.716), and their interaction was not significant (*F*_(2,46)_ = 0.244, *p* = 0.785, $\eta_{\text{p}}^{2}$ = 0.011). From the results of the ANOVA for RT, similarly, the main effect of intervention was not significant (*F*_(1,23)_ = 0.352, *p* = 0.559, $\eta_{\text{p}}^{2}$ = 0.015), the main effect of condition was significant (*F*_(2,46)_ = 12.140, *p* < 0.001, $\eta_{\text{p}}^{2}$ = 0.346), and their interaction was not significant (*F*_(2,46)_ = 1.324, *p* = 0.276, $\eta_{\text{p}}^{2}$ = 0.054). All behavioral data were affected by condition factor only. The results following multiple comparisons using Holm’s sequentially rejective Bonferroni method identified that *d′* under the Word condition was significantly higher than the Cross (*t*_(23)_ = 4.118, *p* < 0.001, *d* = 0.492) and Within condition (*t*_(23)_ = 8.775, *p* < 0.001, *d* = 1.643). Further, *d*′ under the Cross condition was higher than that for the Within condition (*t*_(23)_ = 8.053, *p* < 0.001, *d* = 1.113). RTs under Word (*t*_(23)_ = 3.623, *p* = 0.003, *d* = 0.291) and Cross conditions (*t*_(23)_ = 4.979, *p* < 0.001, *d* = 0.291) were significantly faster than the Within condition; however, there was no significant difference between the Word and Cross condition regarding RT (*t*_(23)_ = 0.570, *p* = 0.574, *d* = 0.032).

**Figure 4:**
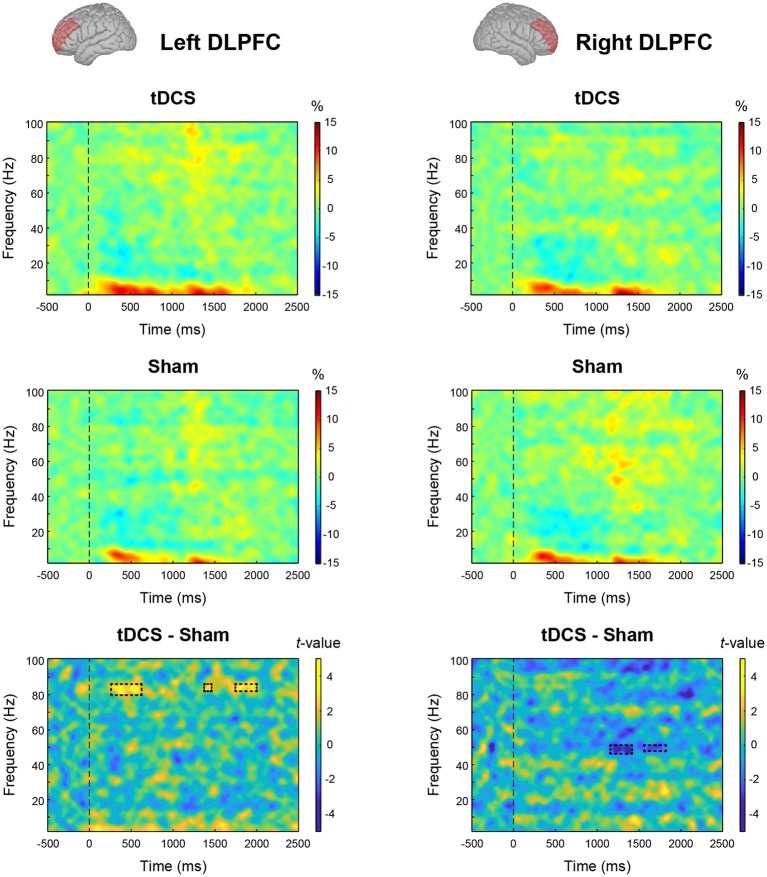
Effect of intervention on oscillatory cortical activity: event-related spectral perturbation (ERSP) plots from the results of time-frequency analysis are given for the tDCS condition and Sham condition in the left and right dorsolateral prefrontal cortex (DLPFC). The bottom panels show the results of the permutation *t*-test (tDCS—Sham). The rectangle regions surrounded by a dotted line indicate significant event-related synchronization (ERS) or desynchronization (ERD) with false discovery rate (FDR) correction (*p* < 0.05).

**Figure 5:**
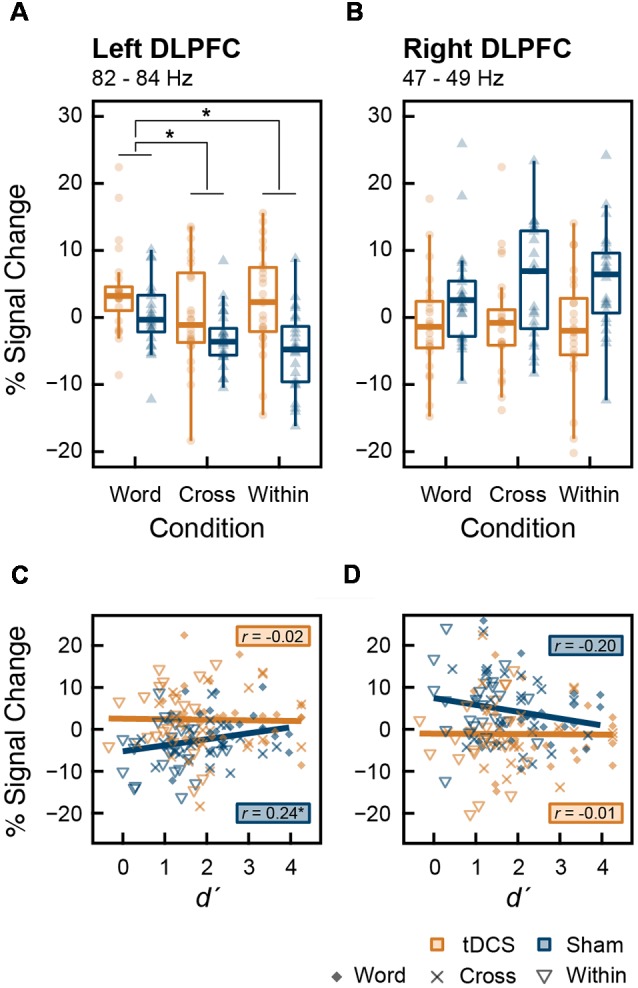
Box plots with individual participant data of percent signal change in **(A)** the left DLPFC and **(B)** the right DLPFC. Data from the left DLPFC were extracted from 270 to 600 ms at 82–84 Hz, and data from the right DLPFC were extracted from 1,180 to 1,400 ms at 47–49 Hz, during which tDCS had significant effects. Stars denote the significance at *p* < 0.05; however, the stars indicating the significant main effect of intervention are omitted. Scatter plots **(C,D)** show the correlation between *d*′, indicating working memory (WM) capacity, and percent signal change that appeared above in the **(A)** left and **(B)** right DLPFC, respectively. The results of correlation analysis (Pearson’s correlation coefficient, *r*) at each intervention are shown in **(C,D)**.

We tested the main effect of intervention on MEG data. From the results of the permutation *t*-test on time-frequency data, tDCS increased high-gamma band power (82–84 Hz) in the left DLPFC from 270 to 600 ms and 1,750–2,000 ms after stimulus onset. In the right DLPFC, tDCS significantly reduced gamma band power in 47–49 Hz band from 1,180 to 1,400 ms and at 49 Hz from 1,610 to 1,720 ms (Figure 4). To explore this result in more depth, we analyzed the data where tDCS had a significant effect on high-gamma band ERS or gamma band ERD using two-way ANOVA. In the left DLPFC at 82–84 Hz, there were significant main effects of intervention (*F*_(1,23)_ = 19.461, *p* < 0.001, $\eta_{\text{p}}^{2}$ = 0.458) and condition (*F*_(2,46)_ = 5.541, *p* = 0.007, $\eta_{\text{p}}^{2}$ = 0.194) on high-gamma band ERS. Their interaction was not significant (*F*_(2,46)_ = 1.579, *p* = 0.217, $\eta_{\text{p}}^{2}$ = 0.064). The results following multiple comparisons showed that percent signal change under the Word condition was significantly higher than that under the Cross (*t*_(23)_ = 2.655, *p* = 0.028, *d* = 0.501) and Within (*t*_(23)_ = 3.229, *p* = 0.011, *d* = 0.218) conditions (Figure 5A). In the right DLPFC at 47–49 Hz, there was a significant main effect of intervention on gamma band ERD (*F*_(1,23)_ = 15.048, *p* < 0.001, $\eta_{\text{p}}^{2}$ = 0.396), and no significant main effect of condition (*F*_(2,46)_ = 0.367, *p* = 0.645, $\eta_{\text{p}}^{2}$ = 0.016) and no interaction (*F*_(2,46)_ = 0.582, *p* = 0.563, $\eta_{\text{p}}^{2}$ = 0.025; Figure 5B).

There was a significant correlation between *d*′ and percent signal change in the high-gamma band oscillation in the left DLPFC after the sham stimulation (*t*_(70)_ = 2.101, *r* = 0.244, *p* = 0.039). There were no other significant correlations (Figures 5C,D). Furthermore, in each ROI and in each intervention, percent signal change data were divided into the three groups corresponding to Word, Cross, and Within conditions; we then conducted correlation analyses in each group (2 ROIs × 2 interventions × 3 conditions). No significant correlations were found within these groups (*p* > 0.05).

**Figure 6:**
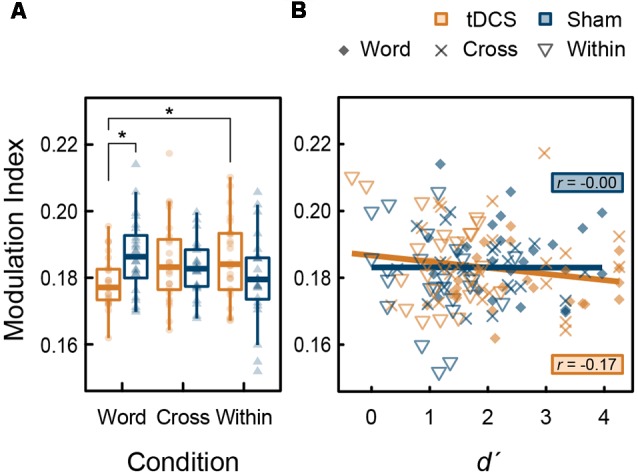
**(A)** Box plots with individual participant modulation index (MI) data showing the strength of phase-amplitude coupling (PAC) in the left DLPFC: stars denote significant difference at *p* < 0.05. **(B)** Scatter plots showing the correlation between *d*′, and MI.

Further analyses were performed to explore the phase-amplitude coupling (PAC) between high-gamma band and theta bands. We also analyzed the modulation index (MI) showing the strength of theta (4–7 Hz) phase and high-gamma (82–84 Hz) amplitude coupling in the left DLPFC within the time of interest, in which tDCS significantly increased high-gamma band power (270–600 ms). In this time window, task-related gamma-band oscillations were present in this region. An increase of MI indicates a phase-dependent increase in amplitude (Canolty et al., 2006; Tort et al., 2010). From the ANOVA results for the MI, the main effect of intervention (*F*_(1,23)_ < 0.001, *p* = 0.987, $\eta_{\text{p}}^{2}$ < 0.001) and condition (*F*_(2,46)_ = 0.212, *p* = 0.810, $\eta_{\text{p}}^{2}$ = 0.009) were not significant; however, their interaction was significant (*F*_(2,46)_ = 5.574, *p* = 0.007, $\eta_{\text{p}}^{2}$ = 0.195). The simple main effect of intervention in the Word condition was significant (*F*_(1,23)_ = 8.819, *p* = 0.007, $\eta_{\text{p}}^{2}$ = 0.277), but those in the Cross (*F*_(1,23)_ = 0.492, *p* = 0.490, $\eta_{\text{p}}^{2}$ = 0.021) and the Within condition (*F*_(2,46)_ = 1.956, *p* = 0.175, $\eta_{\text{p}}^{2}$ = 0.078) were not significant. Regarding tDCS intervention, the condition factor was significant (*F*_(2,46)_ = 3.640, *p* = 0.034, $\eta_{\text{p}}^{2}$ = 0.137). At that level, MI in the Word condition was significantly lower than the Within condition (*t*_(23)_ = 3.335, *p* = 0.009, *d* = 0.715) following a *post hoc* *t*-test using the Holm’s sequentially rejective Bonferroni method. In summary, the significant reduction effect of tDCS on the MI was found in the Word condition (Figure 6A). There was no significant correlation between *d*′ and PAC (tDCS: *t*_(70)_ = −1.492, *r* = −0.176, *p* = 0.140; Sham: *t*_(70)_ = −0.010, *r* = −0.001, *p* = 0.992; Figure 6B). Furthermore, no significant correlations were found with the groups (*p* > 0.05).

## Discussion

We found that offline anodal tDCS over F3 did not improve WM performance in accuracy and speed, partially rejecting our hypothesis (Figure 3). Despite the lack of behavioral changes, tDCS significantly induced high-gamma band ERS (82–84 Hz) in the left DLPFC and gamma band ERD (47–49 Hz) in the right DLPFC (Figure 4). At first, we found that tDCS significantly enhanced high-gamma band power regardless of the condition, because the interaction (intervention × condition) was not significant. However, the main effect of condition was significant, and the Word condition had a higher power than the two other conditions. This implies that high-gamma band power in the left DLPFC could be responsible for activation of verbal WM rather than a domain-general updating ability. Given this, it may be possible that WM capacity does not increase, even if tDCS activated verbal WM, in the Cross and Within conditions, in which colored rectangles were visually remembered. Furthermore, we found a significant positive correlation between the high-gamma band power and WM capacity (*d*′) after the sham stimulus. However, when the data were divided into groups corresponding to the three conditions, group-wise correlations were not significant. Accordingly, the relationship between high-gamma power and WM capacity was spurious, which could be explained by the nature of the task condition. In other words, high-gamma oscillation in the left DLPFC might not affect WM capacity, and it could be altered by the items to be remembered. Our findings also raise the possibility that there are optimal frequencies for updating verbal WM as a mental rehearsal system. During 3-back task, tDCS induced oscillations of a higher frequency than the frequency band (30–45 Hz) known to be effective for the 2-back task accuracy (Hoy et al., 2015a). High-gamma ERS over 50 Hz in the left DLPFC has also been observed in language-related tasks, such as a verb generation task (Hashimoto et al., 2017) and an object naming task (Babajani-Feremi et al., 2014). Another possibility is that the relationship between gamma band power and WM capacity has an “inverted-U” shape, much like that of dopamine and WM (Takahashi et al., 2008). Healthy adults might have an appropriate level of gamma band activity, and tDCS could have a smaller impact on WM capacity than it might in patients with cognitive impairment, whose gamma band power is decreased.

We also observed significant gamma band ERD in the right DLPFC after cathodal tDCS over F4, whereas the effect of condition and the interaction was not significant (Figure 5B). In addition, gamma band power in the right DLPFC was not correlated with WM capacity (Figure 5D). The right DLPFC has been suggested to be responsible for executive function inhibitory control during a Stroop task (Vanderhasselt et al., 2009). The ERD observed in our study seems not to be important for updating ability, verbal WM, or items to remember, because no significant result was found.

From our results, it is still unclear why the tDCS-induced gamma oscillation did not affect WM capacity. There is a possibility that increasing high-gamma band oscillations which do not interact with the lower-band rhythm may not align with improving WM capacity (Turi et al., 2018). From a local field potential study, when the memory system holds multiple items, the population of neurons in the PFC of a rhesus monkey shows phase-dependent activity (Siegel et al., 2009). In human studies, high-gamma (80–150 Hz) amplitude couples to the theta (4–8 Hz) and alpha (8–12 Hz) trough recorded by electrocorticogram; in particular, during several verbal tasks, theta-gamma coupling was prominent in the left DLPFC (Voytek et al., 2010). The MI (Canolty et al., 2006), indicating theta-gamma PAC measured by EEG, has been shown to be greater in healthy adults than patients with mild cognitive impairment or Alzheimer’s dementia during the 2-back task (Goodman et al., 2018). These studies suggest that the complex waves where gamma-band amplitude is coupled to theta-band phase could convey sequential information necessary to perform n-back tasks (Roux and Uhlhaas, 2014).

We found the significant interaction of tDCS and task condition in theta-gamma PAC during the verbal 3-back task (Figure 6A). Indeed, anodal tDCS induced greater high-gamma band power in the left DLPFC (Figure 5A); however, theta-gamma PAC was not affected, or rather reduced, during the task which recruits the left DLPFC. Considering with high-gamma band oscillation mentioned above (Figure 5A), it is possible that, in the Word condition, the decrease in PAC canceled out the enhancement of the high-gamma band power induced by tDCS, which might have activated the verbal WM. While gamma band ERS in the left DLPFC is known to be positively correlated with WM capacity (Hoy et al., 2015a, 2016), the timing of emergence of gamma-band oscillation may also play an important role. One transcranial alteration current stimulation (tACS) study reported that gamma band tACS did not improve WM capacity in patients with schizophrenia (Hoy et al., 2015b). Future studies should aim to uncover the most effective timing of gamma band oscillations for WM capacity in more detail. Moreover, we found no significant correlation between PAC and WM capacity (Figure 6B). Similar to the high-gamma ERS induced by tDCS in this experiment, the frequency of PAC might be also important for WM capacity. In conclusion, our findings provide neurophysiological evidence that the effect of tDCS on WM capacity is not always robust.

Our study has some limitations from the inherent nature of the n-back task. For estimating WM capacity, the n-back task is useful; however, memory functions, such as encoding, maintenance and retrieval, are not clearly distinguishable in time. During the time of interest (270–600 ms), a new item is encoded into WM storage and is compared with the stored item simultaneously. In addition, the pre-stimulus baseline period in a trial may also be the end section of the previous trial as trials were presented continuously. Therefore, baseline correction processes may affect the values in the latter time period of a trial. If we reveal the effect of tDCS on the memory process in detail, memory tasks that have a pre-trial baseline period and distinguish between encoding, maintenance, and recognition, such as a reading span task (Daneman and Carpenter, 1980; Osaka and Osaka, 1992) should be used. Furthermore, a WM task that can overcome the immediate learning effect, introducing a pre-post design for each day would increase the statistical power.

## Ethics Statement

The Ethics Committee of Kanazawa University approved this study, which conformed to the tenets of the Declaration of Helsinki (UMIN Clinical Trials Registry: UMIN000021058).

## Author Contributions

TI, TT, and HH contributed to the conception and design of the study. TI, TT, HH, and DS collected the data. TI performed the statistical analysis and wrote the first draft of the manuscript. All authors contributed to manuscript revision, read and approved the submitted version.

## Conflict of Interest Statement

The authors declare that the research was conducted in the absence of any commercial or financial relationships that could be construed as a potential conflict of interest.

## Footnotes

^1^http://surfer.nmr.mgh.harvard.edu/

^2^http://cran.r-project.org/
