# Supplementary material for: Anodal Transcranial Direct Current Stimulation Induces High Gamma-Band Activity in the Left Dorsolateral Prefrontal Cortex During a Working Memory Task: A Double-Blind, Randomized, Crossover Study
Source: Front Hum Neurosci. 2019 Apr 24;13:136. doi: 10.3389/fnhum.2019.00136 (PMC6491895; doi:10.3389/fnhum.2019.00136)
Supplement: Supplementary file 1 [file Table_1.DOCX]

Supplementary Material

# Supplementary Table 1

**Supplementary Table 1.** Color coordinates according to CIE1931 and the result of color naming task: color names were reported in Japanese. *N* shows the number of participants who reported the color name.

|  | *Y* | *x* | *y* | Color naming (*N*) |  |
| --- | --- | --- | --- | --- | --- |
| Color group 1: purple, red, orange | | | | | |
| Cross | 28.99 | 0.2953 | 0.1845 | purple (24) |  |
|  | 37.18 | 0.6108 | 0.3581 | red (23), yellow (1) |  |
|  | 77.71 | 0.5060 | 0.4617 | orange (16), yellow (7), red (1) |  |
| Within | 36.43 | 0.5454 | 0.3202 | red (15), pink (9) |  |
|  | 37.18 | 0.6108 | 0.3581 | red (21), orange (3) |  |
|  | 48.27 | 0.5951 | 0.3832 | orange (20), red (4) |  |
| Color group 2: orange, yellow, yellow green | | | | | |
| Cross | 77.71 | 0.5060 | 0.4617 | orange (16), yellow (7), red (1) |  |
|  | 208.71 | 0.4064 | 0.5483 | yellow (19), yellow green (5) |  |
|  | 125.05 | 0.3555 | 0.5801 | yellow green (15), yellow (5), green (4) |  |
| Within | 170.54 | 0.4272 | 0.5304 | yellow (23), orange (1) |  |
|  | 208.71 | 0.4064 | 0.5483 | yellow (22), orange (1), yellow green (1) |  |
|  | 165.75 | 0.3881 | 0.5624 | yellow (18), yellow green (6) |  |
| Color group 3: yellow green, green, light blue | | | | | |
| Cross | 125.05 | 0.3555 | 0.5801 | yellow green (15), yellow (5), green (4) |  |
|  | 61.31 | 0.2962 | 0.5016 | green (23), yellow green (1) |  |
|  | 70.95 | 0.2244 | 0.2947 | light blue (19), blue (5) |  |
| Within | 58.17 | 0.3294 | 0.5830 | green (19), yellow green (5) |  |
|  | 61.36 | 0.2962 | 0.5015 | green (23), yellow green (1) |  |
|  | 63.08 | 0.2641 | 0.4115 | green (19), light blue (2), blue (2), yellow green (1) |  |
| Color group 4: light blue, blue, purple | | | | | |
| Cross | 70.95 | 0.2244 | 0.2947 | light blue (19), blue (5) |  |
|  | 38.12 | 0.1672 | 0.1048 | blue (19), purple (5) |  |
|  | 28.99 | 0.2953 | 0.1845 | purple (24) |  |
| Within | 40.34 | 0.1773 | 0.1654 | blue (15), light blue (9) |  |
|  | 38.12 | 0.1672 | 0.1048 | blue (19), purple (3), light blue (2) |  |
|  | 31.94 | 0.1751 | 0.0845 | purple (16), blue (8) |  |
| Background | 126.47 | 0.3136 | 0.3286 |  |  |
